# Micro Three-Dimensional Neuronal Cultures Generate Developing Cortex-Like Activity Patterns

**DOI:** 10.3389/fnins.2020.563905

**Published:** 2020-10-02

**Authors:** Yixuan Ming, Md Fayad Hasan, Svetlana Tatic-Lucic, Yevgeny Berdichevsky

**Affiliations:** ^1^Department of Electrical & Computer Engineering, Lehigh University, Bethlehem, PA, United States; ^2^Department of Bioengineering, Lehigh University, Bethlehem, PA, United States

**Keywords:** 3D culture, neuron, calcium imaging, PDMS, microwell, burst, cortex, drug screening

## Abstract

Studies aimed at neurological drug discovery have been carried out both *in vitro* and *in vivo*. *In vitro* cell culture models have showed potential as drug testing platforms characterized by high throughput, low cost, good reproducibility and ease of handling and observation. However, *in vitro* neuronal culture models are facing challenges in replicating *in vivo*-like activity patterns. This work reports an *in vitro* culture technique that is capable of producing micro three-dimensional (μ3D) cultures of only a few tens of neurons. The μ3D cultures generated by this method were uniform in size and density of neurons. These μ3D cultures had complex spontaneous synchronized neuronal activity patterns which were similar to those observed in the developing cortex and in much larger 3D cultures, but not in 2D cultures. Bursts could be reliably evoked by stimulation of single neurons. Synchronized bursts in μ3D cultures were abolished by inhibitors of glutamate receptors, while inhibitors of GABA_A_ receptors had a more complex effect. This pharmacological profile is similar to bursts in neonatal cortex. Since large numbers of reproducible μ3D cultures can be created and observed in parallel, this model of the developing cortex may find applications in high-throughput drug discovery experiments.

## Introduction

Developing cortex is characterized by bursts of spontaneous synchronized activity of populations of neurons, based on studies performed in pre-term infants and in neonatal rodents (Khazipov and Luhmann, 2006; Egorov and Draguhn, 2013; Luhmann et al., 2016; Arichi et al., 2017). Bursts in somatosensory and visual cortices may be driven by peripheral activity (Colonnese and Khazipov, 2012; Yang et al., 2016). However, cortex may also generate burst activity independently (Garaschuk et al., 2000; Lischalk et al., 2009; Namiki et al., 2013). This activity is important for neuronal maturation and formation of neural circuits, and may play a role in developmental neurological and psychiatric disorders (Kirischuk et al., 2017), some of which are characterized by altered neural activity (André et al., 2010). Experimental models of cortical bursts are therefore of high importance to elucidate burst mechanisms, to determine effects of bursts on neuronal circuits, and to develop therapies for disorders.

Development of therapies, in particular, requires that the model is characterized by high experimental throughput. Phenotypic screens are used in the drug development pipeline to identify promising lead in libraries consisting of hundreds or thousands of compounds (Parsons, 2019). The rapidity with which the effect of a compound on disease phenotype can be assessed determines the throughput of a screen. Current models of cortical bursts include direct *in vivo* measurements of cortical activity in rodent neonates, and *in vitro* measurements of activity in brain slices soon after dissection or after maintaining the slices in an incubator for several days or weeks (Table 1). These models are relatively labor-intensive, and are not scalable to high-throughput phenotypic screens. The challenge is then to develop a scalable model of cortical bursts that captures their salient features.

**TABLE 1:** Burst patterns and drug responses from previous *in vitro* and *in vivo* studies.

**References**	**Experimental details**	**Burst patterns**	**Drug response**
Garaschuk et al., 2000	Ca^2+^ imaging of horizontal slices from P1-2 rats.	Interburst interval (IBI) = 3.65 s, up to 15 s burst duration, single and multi-peak	TTX blocked activity, Glutamatergic inhibitors blocked activity, GABAergic inhibitors did not block
Corlew et al., 2004	Ca^2+^ imaging of horizontal slices from P0 rats	IBI = 1.1 min, 5–15 s burst duration, single and multi-peak	TTX blocked activity
Allene et al., 2008	Ca^2+^ imaging of horizontal slices from E20-P9 rats	Data from P0-3 rats: IBI = 0.41 min, up to 10 s burst duration, single and multi-peak	Glutamatergic inhibitors blocked activity, GABAergic inhibitors did not block
Namiki et al., 2013	Horizontal slices from P1-6 rats	IBI = 0.42 min, up to 10 s burst duration, single and multi-peak	TTX blocked activity, Glutamatergic inhibitors blocked activity, GABAergic inhibitors did not block
Adelsberger et al., 2005	Optical fiber recording from P3-4 mouse	IBI = 0.44 min (from examples), 3.8 ± 0.1 s burst duration (longest example is 16 s), single and multi-peak	N.A.
Conhaim et al., 2011	Organotypic cultures from E17 mice, evaluated for 14 days *in vitro*	IBIs vary widely. Shortest: 0.25 min. Up to 9.3 s burst duration, single and multi-peak	Glutamatergic inhibitors inhibited, but most effective (complete block) after DIV 9, GABAergic inhibitors blocked until DIV 8, then stimulated
Golshani et al., 2009	Ca^2+^ imaging of mice from P4 to adulthood	In neonates, IBI = 0.63 min (from examples), up to 10.6 s burst duration, single and multi-peak	N.A.
Sheroziya et al., 2009	Electrical recordings in horizontal slices of P1-13 rats	IBI = 0.28 min for P5-7, 3.5 ± 1.2 s burst duration at P5-7; 4.8 ± 2.5 s at P11-13, single and multi-peak	TTX blocked activity, Glutamatergic inhibitors blocked activity, GABAergic inhibitors elicited discharges at P5-7

**FIGURE 1:**
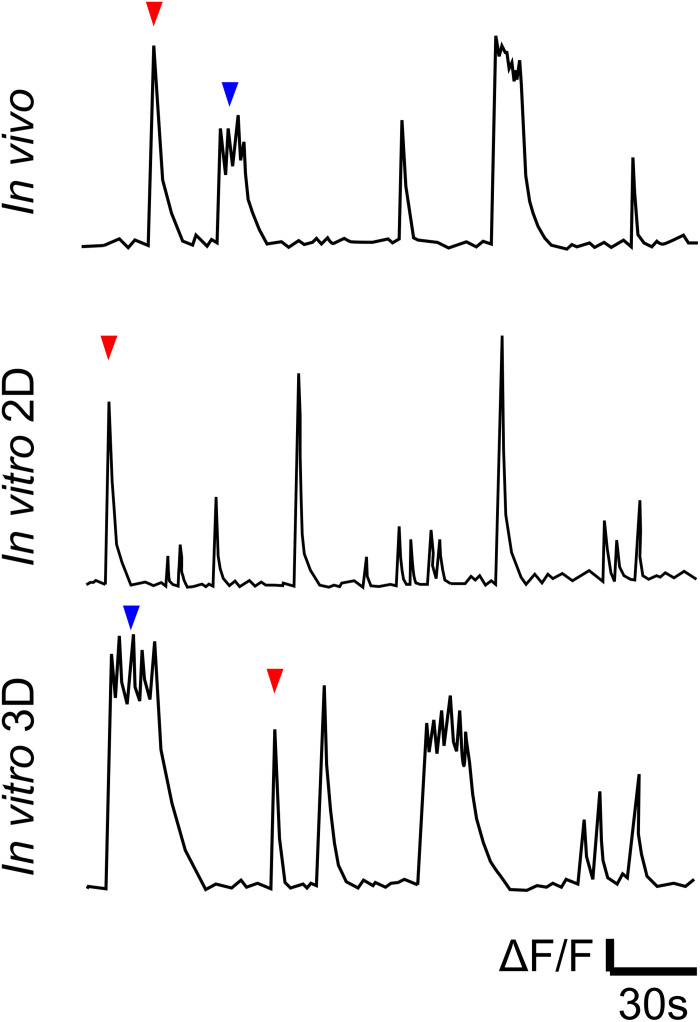
Cartoon illustration of Ca^2+^ traces of *in vivo* (representative of data in references in Table 1), *in vitro* 2D and *in vitro* 3D models (Hasan et al., 2019). Traces illustrate qualitative differences between spontaneous bursts in 2D cultures, on the one hand, and *in vivo* and in 3D cultures, on the other. Red and blue arrows indicate single- and multi-peak events, respectively.

One possible candidate is the culture of dissociated neonatal rodent neurons. A single dissection generates millions of cortical neurons that can be maintained in a multi-well format, resulting in a high degree of parallelism suitable for high-throughput screens (Cooper et al., 2017). Dissociated neurons plated onto a treated substrate grow axons and dendrites and establish synaptic connections (Hasan and Berdichevsky, 2016). Resulting 2D networks are characterized by the presence of spontaneous activity that includes synchronized population bursts. However, bursts in 2D networks are qualitatively different than bursts *in vivo* or in slice models. When evaluated via Ca^2+^ imaging, 2D bursts appear as single-peak events with a decaying tail, while bursts *in vivo* or in slices are a mix of single-peak and multi-peak events (Figure 1 and Table 1). When evaluated via electrical recordings, 2D bursts are synchronized events with short (<1 s) duration and typically a single-peak population spike (van Pelt et al., 2004; Wagenaar et al., 2006), while synchronized activity in the intact brain or slices includes multi-peak events lasting multiple seconds (Namiki et al., 2013), similar to immature human cortex (Vanhatalo et al., 2005). The presence of multiple population peaks in a burst may be due to the burst wave reflections from network edges, such as borders between different brain regions (Golshani et al., 2009; Namiki et al., 2013). The functional significance of multi-peak bursts may be that they result in significantly longer calcium elevations (Table 1) than single-peak bursts. Longer calcium transients (>10 s in cortical burst models vs. ∼1 s in 2D dissociated cultures) may initiate different intracellular signaling pathways linked to neuronal maturation and circuit formation.

We and others have previously shown that burst activity in three-dimensional (3D) cultures of dissociated neurons is qualitatively different from that in 2D, and is characterized by the presence of prolonged, multi-peak, synchronized bursts (Wevers et al., 2016; Marom et al., 2017; Hasan et al., 2019). One approach to generate 3D cultures is to seed dissociated cells onto 3D scaffolds (Daud et al., 2012; Frega et al., 2014; Huang et al., 2014; Puschmann et al., 2014). However, limitation of this approach is that the spatial arrangement of cells in these 3D models is sparse, contradictory to *in vivo* models where cells are densely packed. An alternative approach is to seed dissociated cells into polydimethylsiloxane (PDMS) wells with diameter ranging from several hundred micrometers to millimeters (Park et al., 2015; Hasan et al., 2019). Three-dimensional cultures generated by this approach are relatively large in terms of physical size and the number of neurons, and are not well-suited for high throughput phenotypic screening. Large number of neurons may not be necessary for burst generation: synchronized domains as small as 100 μm containing < 100 neurons have been reported in slices and *in vivo* (Corlew et al., 2004; Golshani et al., 2009).

Methods of forming 3D cultures smaller than 100 μm have also been established. One of the well-known methods is the hanging drop method, in which dissociated cells are cultured in hanging droplets and form spheroids due to self-aggregation. One major limitation of this method is that it is labor-intensive and/or requires special-maintaining equipment because cells are cultured in droplets of around 20 μL (Tung et al., 2011). Another approach of forming 3D cultures small than 100 μm is to seed cells into microfabricated devices, the sizes of which are on micrometer scale. This approach has been widely used for various applications. For example, microwell arrays fabricated of or coated with non-adhesive materials, such as poly(ethylene glycol) (PEG) and agarose, have been used to study formation of homogeneously sized embryonic bodies (EBs) from murine embryonic stem cells (mESCs) (Karp et al., 2007; Moeller et al., 2008; Sakai et al., 2010; Pasturel et al., 2019), and effect of size on their differentiation (Karp et al., 2007; Jeong et al., 2012). Formation and differentiation of EBs have also been investigated with microwells attached to adhesive substrates (Mohr et al., 2006; Park et al., 2007). Neurospheres formed from murine neural progenitor/stem cells (mNPCs) in PEG microwells have been studied for self-renewal ability and multipotency (Cordey et al., 2008). Microwells and microfluidic devices have also been utilized to form *in vitro* 3D disease models from pancreatic islets cells (Ichihara et al., 2016), hepatocytes (Sakai et al., 2010), human embryonic kidney (HEK) cells (Pasturel et al., 2019), human hepatoblastoma cells (HepG2) (Sakai et al., 2010; Selimović et al., 2011; Ong et al., 2017), and cortical neurons (Choi et al., 2013). Networks formed with neurospheres that are around 100 μm in diameter have been studied (Kato-Negishi et al., 2010), where neurospheres were harvested from PDMS microwells and cultured on polyethyleneimine (PEI)-coated glass plates. Neighboring neurospheres formed random connections and synchronized spontaneous activities were observed. Although 3D cultures of cortical neurons that are less than 100 μm in size have been reported (Kato-Negishi et al., 2010; Choi et al., 2013; Pasturel et al., 2019), little has been done to evaluate the activity patterns. Also, these 3D cultures were connected by sprouting axons into a single network, limiting the throughput of this platform for drug discovery.

In this work, we report a novel method of creating micro-3D (μ3D) cultures of dissociated neurons with dimensions below 100 μm. These μ3D cultures are closely spaced for simultaneous optical evaluation, but are not connected with axons and represent separate, independent networks. We then evaluate activity in μ3D cultures to determine if they model developmental cortical bursting. Our evaluation criteria included burst waveform (single vs. multi-peak), burst duration, and dependence on voltage-gated sodium channels and glutamatergic or GABAergic neurotransmission, as reported for developmental cortical bursts (Table 1).

## Materials and Methods

### SU-8 Master and PDMS Microstructure Fabrication

First, an 80-μm-thick layer of SU-8 2050 (Microchem Corp.) was spun onto a 3-inch silicon wafer at 4,000 revolutions per minute (RPM) for 1 min. Then, the wafer was baked on a hotplate at 65°C for 3 min and 95°C for 6 min. After cooling down to room temperature, the wafer was exposed to ultraviolet (UV) light through a mask for a total exposure energy of 160 mJ ^cm−2^. Next, the wafer was baked on a hotplate at 65°C for 1 min, followed by baking on 95°C for 6 min, and then cooled down to room temperature. After development in SU-8 developer (Microchem Corp.) for 5 min, only SU-8 patterns defined by the mask stayed on the wafer. The SU-8 patterns were rinsed with Isopropyl Alcohol (IPA) and dried with nitrogen. Finally, the SU-8 master was hard baked at 120°C overnight, followed by silanization for 16 h at room temperature in a vacuum desiccator filled with trichloro(1H,1H,2H,2H-perfluorooctyl)silane (Sigma-Aldrich).

**FIGURE 2:**
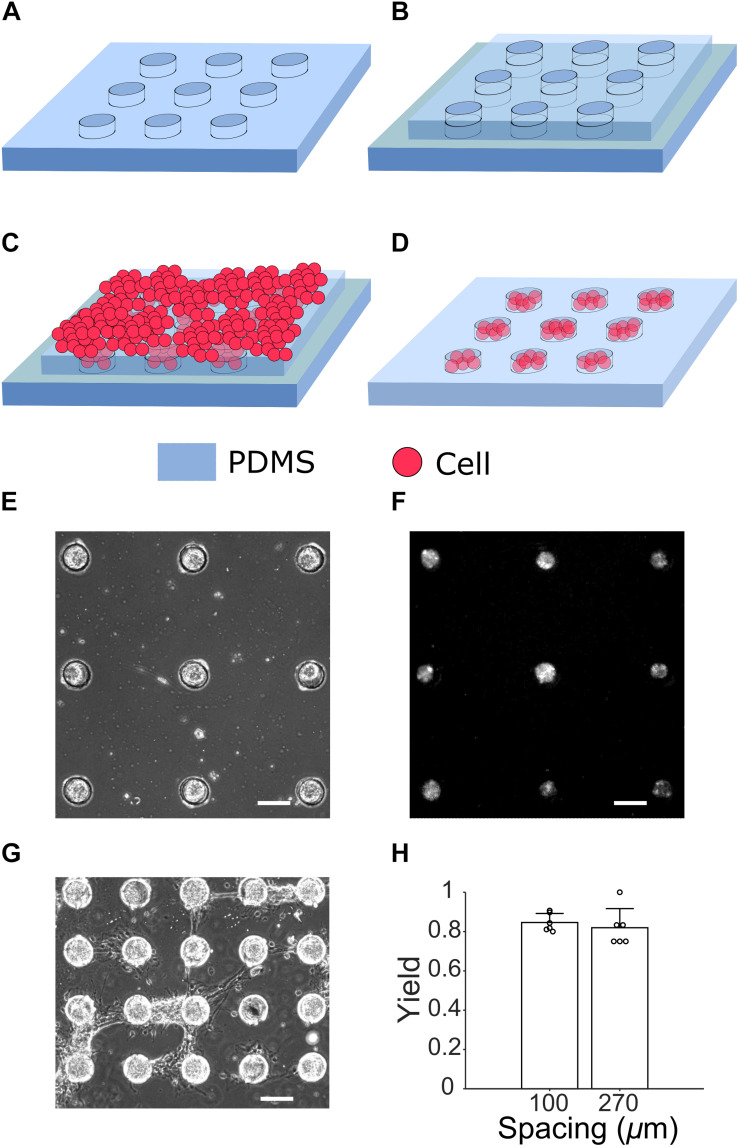
Cell culture steps for generating μ3D cultures. **(A)** A PDMS microstructure to be attached to a PDL-coated glass slide (not shown). **(B)** Under a stereo microscope, an identical but slightly smaller microstructure is aligned to the first layer **(C)** Cells suspended in seeding media are placed on top of the two-layer microstructure. **(D)** After removal of the second layer on DIV 1, cells in PDMS wells form μ3D cultures. A phase contrast micrograph **(E)** and fluorescent micrograph of jRGECO1a expression **(F)** of μ3D cultures on DIV 8 show formation of neuronal clusters in microwells. **(G)** Bridges of axons are formed between neighboring μ3D cultures on DIV 8 when the wells spacing is only 100 μm. Scale bar in **(E–G)**: 100 μm. **(H)** Yields of μ3D cultures for different inter-well spacing. Bars represent the average and error bars are standard deviations. Each circle represents a data point. Student’s *t*-test showed no significance, *p* = 0.56, *n* = 6 field of views from 2 cultures for each spacing.

Polydimethylsiloxane (PDMS) base was mixed with curing agent (Dow Corning Corp.) at 10:1 ratio and left in a vacuum desiccator for 15 min to remove bubbles. The mixture was then spun onto the SU-8 master at 1000 RPM for 3 min and cured at 75°C overnight, leading to an 80 μm thick PDMS layer. PDMS microstructures were carefully peeled off from the master, cut into desired size and sterilized in ethanol. After drying, a piece of PDMS microstructure was carefully lowered onto a coverslip coated with poly-D-lysine (PDL, Sigma-Aldrich) (Figure 2A). A second layer of PDMS microstructure, which is slightly smaller (for the ease of handling), was aligned on top of the first one under a stereo microscope (Figure 2B). The second layer served as a cellular mask and was removed after cell seeding, which prevents cells from attaching to undesired locations. Finally, the structure was placed into a 35-mm-diameter Petri dish, immersed under cell culture media [97.5% Neurobasal-A media (Invitrogen), 2% B27, 1 mM glutaMAX (Gibco) and 30 μg/mL gentamicin (Gibco)] and incubated at 37°C overnight to reduce cytotoxicity.

### Cell Dissociation and Culture

Cortices were obtained from postnatal day 0-1 (p0-1) Sprague-Dawley rat pups (Charles River Laboratories) per the protocol described in Brewer et al. (1993). All animal use protocols were approved by the Institution Animal Care and Use Committee (IACUC) at Lehigh University and were conducted in accordance with the United States Public Health Service Policy on Humane Care and Use of Laboratory Animals. Cortices were then cut and dissociated with papain (Worthington Biochemical Corp.) in Hanks’ balanced salt solution (HBSS, Gibco) for 20 min. The dissociated cortices were washed with fresh media three times. Cells were obtained from the dissociated cortices after trituration and centrifugation. Next, cells were resuspended with cell seeding media [Neurobasal-A, 0.5 mM GlutaMAX, 30 μg/mL gentamicin and 10% Fetal Bovine Serum (FBS, Gibco)] at desired concentration. Method for generating 1300 μm 3D cultures was published elsewhere (Hasan et al., 2019). For 2D cultures, cells were seeded onto a PDL-coated cover slip at 0.5 M/mL. For μ3D cultures, a total number of 0.508 M cells and 20 μL of cell seeding media were added inside the PDMS device described in the previous section (Figure 2C). After 15 min, another 2 mL of cell seeding media was added to the dish. Cells were allowed to settle down for 45 min and then cell seeding media was replaced with 2 mL fresh culture media (see section “SU-8 Master and PDMS Microstructure Fabrication” for the recipe). One day after cell plating, cellular mask was carefully removed (Figure 2D). Cell culture was incubated at 37°C with 5% CO_2_, and half of the culture media was replaced twice per week.

### Immunocytochemistry (ICC) and Cell Counting

Cell cultures were fixed at room temperature with 4% paraformaldehyde (PFA, Electron Microscopy Science) in phosphate buffered saline (PBS, Sigma-Aldrich) for 1 h, and then permeabilized in 0.3% Triton X-100 in PBS for 15 min. Next, cultures were blocked with 10% goat serum in 0.05% Triton X-100 in PBS. Primary antibodies anti-MAP2 (1:2000 dilution, BioLegend), SMI312 (anti-Neurofilament Marker antibody, 1:500 dilution, BioLegend), anti-GFAP (Anti-Glial Fibrillary Acidic Protein Antibody, 1:500 dilution, Sigma-Aldrich) or Anti-GAD67 (1:500 dilution, Sigma-Aldrich) were added to the culture which was then kept at 4°C for 3 days. After wash, secondary antibodies were added accordingly. The culture was kept at 4°C for another 3 days. Finally, DAPI (Invitrogen) was added after wash. Cultures were then washed, mounted and imaged on a confocal microscope (Zeiss LSM 510 META, Germany). Three dimensional reconstruction of cell cultures was performed in ZEN lite (Zeiss).

Cell counting was done manually by analyzing confocal images in ImageJ (Schneider et al., 2012). DAPI-positive objects were counted as cells with tiny and bright ones excluded. Objects that were MAP2-positive (MAP2^+^) or jRGECO1a-expressing and co-localized with a cell count, were marked as neurons. Curve fitting of number of cells to seeding concentration and drug response of phenytoin were done in MATLAB using the curve fitting toolbox. GFAP-positive (GFAP+) cells and (GAD67+) cells were counted as astrocytes and GABAergic neurons, respectively.

### Whole Cell Recording

Whole cell recordings were performed on DIV 18–20. Upright microscope (Olympus BX51WI, Olympus Optics, Japan) equipped with infrared-differential interference contrast optics was used to locate neurons in μ3D cultures. The recordings were conducted at 32–34°C, and the resistance of the recording pipette (1.5 mm borosilicate glass, Sutter Instruments) was 4 to 7 MΩ. The cultures were held in a perfusion chamber with constant flow of recording solution composed of: 120 mM NaCl, 3.5 mM KCl, 1.3 mM CaCl_2_, 0.9 mM MgCl_2_, 25 mM NaHCO_3_, 1.23 mM NaH_2_PO_4_, and 10 mM glucose, bubbled with 5% CO_2_ 21% O_2_, and balanced with N_2_. In some experiments, 3 mM kynurenic acid (KYNA) was added to recording solution to prevent network bursts. The recording pipette was loaded with intracellular solution containing 130 mM K-gluconate, 5 mM KCl, 4 mM ATP-Mg, 0.3 mM GTP, 10 mM HEPES, and 10 mM phosphocreatine (pH 7.2, adjusted with KOH). Up to 2 cells were patched from each culture. Current-voltage curves were recorded in current clamp mode using MultiClamp 700B (Molecular Devices) and acquired at sampling frequency of 20 kHz through DigiData 1550B and pCLAMP 10 software (Molecular Devices). The data was analyzed in pCLAMP 10, without correction for liquid junction potential.

### Optical Recording

To record neural activity optically, cells were infected on day *in vitro* (DIV) 1 with adeno-associated virus (AAV) to express red genetically encoded calcium indicator for optical recording (jRGECO1a) under Syn promoter [AAV9, pAAV.Syn.NES-jRGECO1a.WPRE.SV40, which was a gift from The Genetically Encoded Neuronal Indicator and Effector Project (GENIE) & Douglas Kim (Addgene viral prep # 100854-AAV9^1^; RRID:Addgene_100854)]. Dynamic fluorescence changes of jRGECO1a caused by neuronal activities could be observed from around DIV 8. To record the fluorescence changes, the dish containing infected cell culture was placed into a mini incubator chamber (Bioscience Tools). The chamber was maintained at 37°C and supplied with humidified gas mixture (5% CO_2_, 21% O_2_ and balanced N_2_, Airgas). Fluorescence intensity was recorded for 12 min with an inverted microscope (Olympus).

### Burst Detection and Analysis

To extract fluorescence intensities from optical recordings, regions of interest were drawn around the whole μ3D culture or single cells in 2D culture. Mean gray value *F* of each 3D culture and each cell in 2D cultures was measured using ImageJ (Schneider et al., 2012). Baseline *F*_*0*_ of each recording was obtained using MATLAB and the algorithm described in Eilers and Boelens (2005). Fluorescence change, Δ*F*/*F*, was calculated by:

$$\Delta \,\text{F}/\text{F}=\left(\text{F}-{\text{F}}_{0}\right)/{\text{F}}_{0}$$

A threshold of mean plus two times standard deviation of the baseline was set to detect activities. When the fluorescence surpassed the threshold, it was considered as the start of an activity and the activity was considered to end when the fluorescence dropped below the threshold.

For μ3D cultures and large 3D cultures, fluorescence changes were interpreted as network-wide synchronized neuronal activities or population bursts (referred as bursts). For 2D cultures, activities of single cells were detectable. Only activities involving more than 50% of the total neurons within the network were considered as population bursts (or bursts) and used for data analysis.

### Pharmacological Experiments

Cultures were recorded in regular culture media (vehicle) first. Next, they were incubated for 30 min in culture media containing kynurenic acid (KYNA, 3 mM, Sigma-Aldrich) or bicuculline (10 μM, Tocris) and recorded in the same media. Next, cell cultures were washed three times with fresh regular media and incubated for 2 h with regular culture media before the final (wash-out) recording.

For the APV [D-(-)-2-Amino-5-phosphonopentanoic acid, Tocris] and NBQX (2,3-Dioxo-6-nitro-1,2,3,4-tetrahydrobenzo[f]quinoxaline-7-sulfonamide, Tocris) experiment, cultures were recorded in regular culture media (vehicle) first. Then, they were incubated for 30 min with culture media containing APV (50 μM) and recorded in the same media. Next, cultures were incubated for another 30 min with culture media containing APV (50 μM) and NBQX (10 μM) and recorded. Finally, cultures were washed three times with fresh culture media, incubated for 2 h with regular culture media and recorded for the wash-out recording.

To measure the response of μ3D cultures to phenytoin (10, 20, 50, 100, and 150 μM, Sigma-Aldrich), cultures were first recorded in regular culture media. Then, cultures were incubated for 1 h in culture media containing the lowest concentration of phenytoin. After the incubation, they were recorded. Next, culture medium was changed to medium with next higher concentration of phenytoin and cultures were recorded after another 1 h of incubation. The process was repeated until the highest concentration of phenytoin was tested. Finally, cultures were washed three times with fresh media and recorded for the final (wash-out) recording. Data were collected for analysis from cultures that were active for both vehicle and wash-out recordings.

## Results

When microwells were distributed sparsely (270 μm spacing in Figure 2E), they enabled good confinement for cells and neurites. However, when microwells were too close to each other, neurites of cell clusters were able to climb over microwell walls and formed bridges which connected neighboring cell clusters (100 μm spacing in Figure 2G). The bridges of neurites were observed starting from DIV 6 when spacing between microwells was 100 μm. The yield (% of microwells with μ3D neuron cluster) was calculated based on phase contrast pictures taken on DIV 6 (Figure 2H). There is no significant difference between average yields of 100-μm-spacing (0.846) and 270-μm-spacing (0.819) microwells. High yield is evidence that this method generates μ3D cultures reliably and robustly.

**FIGURE 3:**
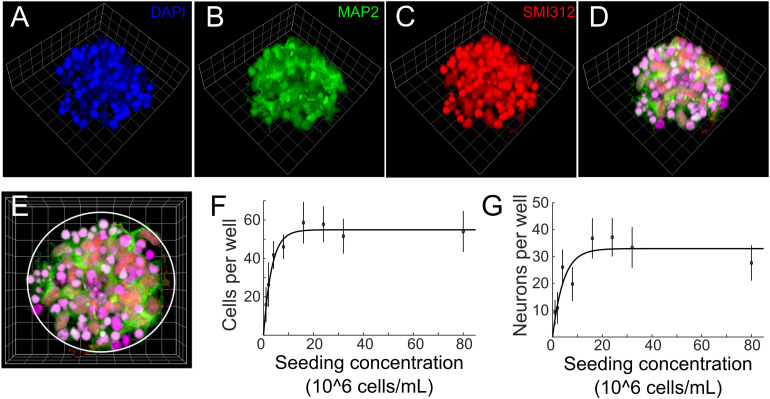
**(A–D)** 3D reconstruction of a cell cluster in a PDMS well. Side length of the reference squares is 7.5 μm. Cells were fixed on DIV 3 and stained with **(A)** DAPI (blue, nuclei), **(B)** anti-MAP2 (green, soma and dendrites of neurons) and **(C)** SMI312 (red, soma and axons of neurons). Colocalization of green, red and blue is displayed as magenta in panel **(D)**. **(E)** Top view of the μ3D culture. White circle denotes boundary of the PDMS well. Average number of cells **(F)** and neurons **(G)** per well at different seeding concentrations. Error bars are standard deviations. *n* = 7 μ3D cultures for each seeding concentration. Solid lines are curves fitted to exponential functions using least absolute residuals (LAR) method. *r*^2^ = 0.96, *p* = 1.61 ×10^−5^ for **(F)** and *r*^2^ = 0.77, *p* = 4.1 ×10^−3^ for **(G)**.

To characterize the morphology and quantify number of cells and neurons of μ3D cultures, cell cultures were fixed on DIV 3, co-stained with anti-MAP2 for soma and dendrites of neurons, SMI312 for axons of neurons and DAPI for nuclei (Figures 3A–E), and imaged with a confocal microscope. The seeding concentration for the μ3D culture shown in Figures 3A–E is 2.4 × 10^∧^7 cells/mL. Cell counting was done manually in ImageJ. Since small and bright DAPI positive objects indicate shrunk nuclei of unhealthy cells (Casiano et al., 1998), only cells with morphologically normal nuclei were included in the cell count. MAP2^+^ objects were counted as neurons if they contained a morphologically normal nucleus. Number of cells and neurons per well and the corresponding seeding concentration are shown in Figures 3F,G, in which circles represent data points and lines are curves fitted to exponential functions. Number of cells per well increased with the seeding concentration and reached the maximum of 58.6 cells per well at seeding concentration of 1.6 × 10^∧^7 cells/mL. Number of cells did not change significantly when seeding concentration exceeded 1.6 × 10^∧^7 cells/mL, likely because the maximum capacity of the PDMS microwell was reached. Similarly, number of neurons per well increased with the seeding concentration and reached the maximum of around 37.1 neurons per well at seeding concentration of 1.6 × 10^∧^7 cells/mL (Figure 3G). Aiming at efficiently generating μ3D cultures containing maximum number of neurons, the seeding concentration was then set to 1.6–2.4 × 10^∧^7 cells/mL for PDMS microwells of 70 μm diameter and 80 μm height. At this seeding concentration, the ratio of standard deviation to average number of neurons in the culture is 0.19, which speaks to the reproducibility of this culture method. If the volume of PDMS wells increases, it is expected that the size of μ3D cultures and average number of cells and neurons would also increase. Thus, different sizes of μ3D cultures may be generated by varying the volume of the PDMS microwells and the corresponding seeding concentration.

**FIGURE 4:**
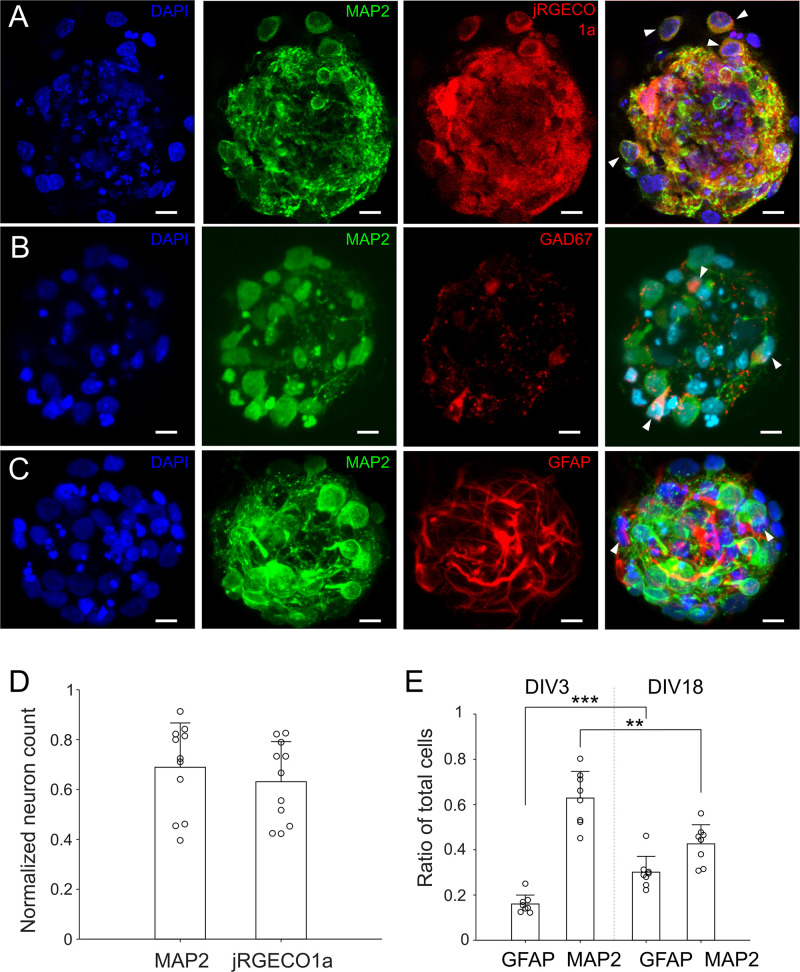
Neurons and astrocytes in μ3D cultures. **(A)** Cultures were infected by AAV9 with jRGECO1a on DIV 1, fixed on DIV 14 and stained with DAPI (blue, nuclei) and anti-MAP2 (green, soma and dendrites of neurons). On right is the merge of DAPI, anti-MAP2 and jRGECO1a fluorescence, with white arrows indicating MAP2^+^ cells (neurons) expressing jRGECO1a. **(B)** Cultures were fixed on DIV 18 and stained with DAPI, anti-MAP2 and anti-GAD67 (GABAergic neuron marker). White arrows in the merged image indicate GABAergic neurons (MAP2^+^/GAD67^+^). **(C)** Cultures were fixed on DIV 18 and stained with DAPI, anti-MAP2 and anti-GFAP (astrocyte marker). White arrows in the merged figure indicate examples of astrocytes (MAP2^–^/GFAP^+^). **(A–C)** Are z-stacks of 5–10 μm thick. **(D)** Neuron counts based on anti-MAP2 and jRGECO1a expression, which were normalized by corresponding cell counts (DAPI). Bars represent the averages and error bars the standard deviations. Each circle represents a μ3D culture. Student’s *t*-test showed no significance, *p* = 0.43, *n* = 11. Scale bar: 15 μm. **(E)** Ratio of astrocytes and neurons on DIV 3 and 18. Student’s *t*-tests were used to evaluate statistical significance. **p < 0.01 and ***p < 0.001, *n* = 8 cultures for each DIV.

For optically recording neuronal activities, cultures were infected by AAV9 with jRGECO1a on DIV 1 after removal of the cellular mask. Cultures expressing jRGECO1a were stained with anti-MAP2 on DIV 14 (Figure 4A). Neuron counting based on anti-MAP2 staining and jRGECO1a expression yielded similar results (Figure 4D). The average percentage of anti-MAP2 positive neurons that were also expressing jRGECO1a was 93% ± 14.8%, indicating that expression of jRGECO1a was of high efficiency, and that activity of nearly all neurons in culture contributed to the recorded activities. Ratio of GABAergic to glutamatergic neurons was 0.18 ± 0.05 (*n* = 20 cultures, Figure 4B), similar to the ratio reported in earlier, larger *in vitro* 3D culture model (Frega et al., 2014). Astrocytes and their processes were present throughout the μ3d culture volume (Figure 4C) and the ratio of astrocytes to total cells increased from 0.15 ± 0.04 on DIV 3 to 0.30 ±0.07 on DIV18 (Figure 4E), indicating proliferation of astrocytes through time.

**FIGURE 5:**
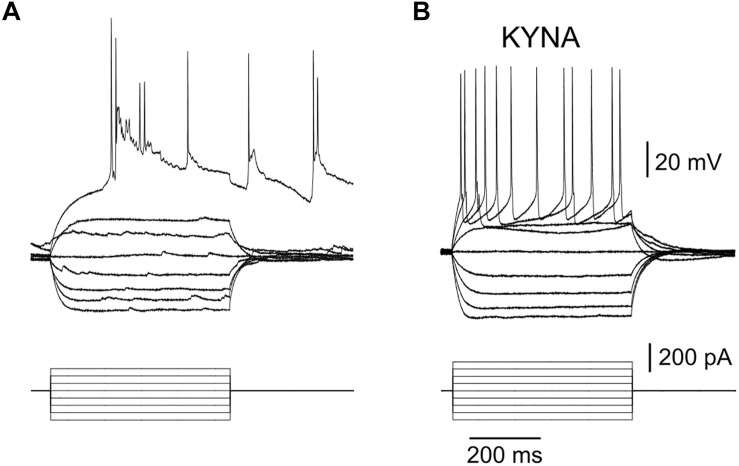
Burst triggered by a single action potential. **(A)** Bottom: injected current pulses (current clamp mode, base injected current is zero), top: recorded voltage shows that 1st evoked AP triggered a burst when culture was in a regular recording solution. Resting membrane potential (baseline of the voltage curves) for this neuron was –65.1 mV. Results are representative of 8 IV curves obtained in 4 neurons. **(B)** No bursts were triggered when 3 mM KYNA was added to the recording solution (resting membrane potential (baseline of voltage curves) was –64.8 mV in this recording), results are representative of *n* = 9 neurons.

IV curves obtained with whole cell recordings of neurons in μ3D cultures in current clamp mode showed that the first action potential evoked with injected current always triggered a network burst (Figure 5A, bursts triggered with 1st evoked action potential in *n* = 8/8 IV curves from 4 neurons in 4 different cultures). When recorded in the presence of 3 mM kynurenic acid (KYNA), there were no network bursts (Figure 5B), and IV curves were typical of cortical neurons *in vitro* (Barral and Reyes, 2016). Input resistance *R*_N_ = 182.6 ± 66.9 MΩ, resting membrane potential *V*_m_ = −66.1 ± 7.6 mV, *τ*_m_ = 23 ± 14 ms, threshold voltage *V*_t_ = −41.2 ± 5.9 mV, threshold current *I*_t_ = 130.6 ± 52.7 pA, action potential (AP) half-width was 1.67 ± 0.78 ms, AP amplitude was 62.0 ± 7.4 mV, afterhyperpolarization voltage was 12.33 ± 4.0 mV, and AP frequency at 400 pA injected current was 24 ± 7.1 Hz, average ± standard deviation values were obtained from *n* = 9 neurons in 8 cultures in the presence of 3 mM KYNA.

**FIGURE 6:**
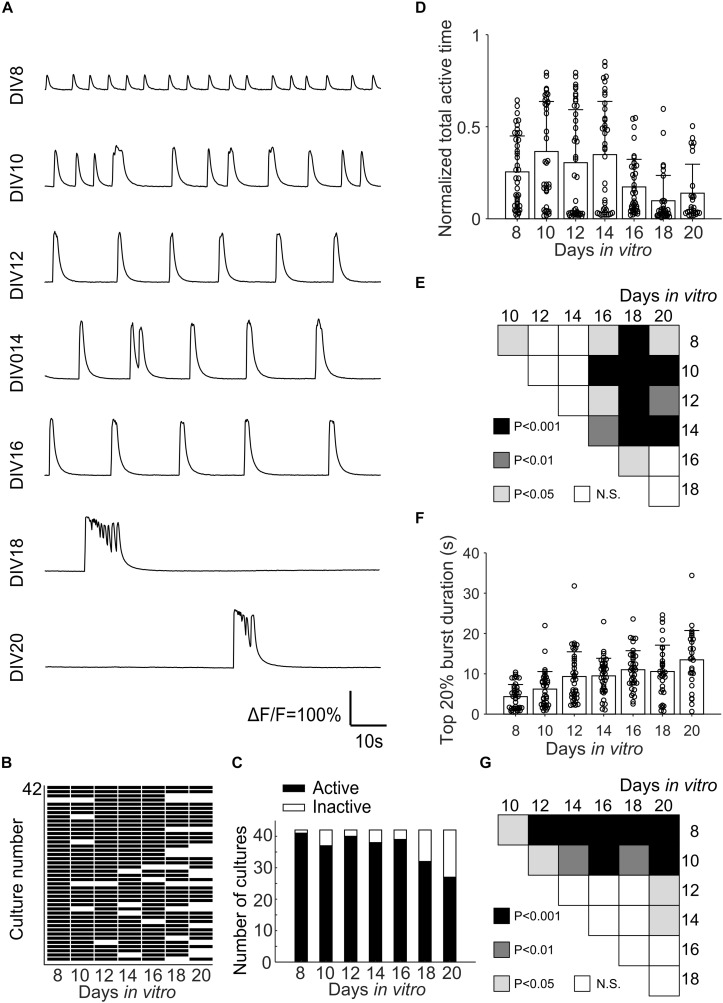
**(A)** Representative Ca^2+^ activity of the same μ3D culture from DIV 8 to DIV 20. **(B)** Map of active and inactive (during 12 min-long recording) μ3D cultures (total culture *n* = 42 from 4 batches) on different DIVs. **(C)** Total active and inactive cultures on different DIVs. Normalized total active time **(D)** and top 20% burst duration **(F)** of μ3D cultures on different DIVs. Students’ t-tests of normalized total active time **(E)** and top 20% burst duration **(G)** between DIVs indicated on x and y axis, N.S. represents no significance. In panels **(D,F)**, bars represent the average and error bars are standard deviations. Each circle represents a culture. *n* = 42 minus number of inactive cultures for each DIV.

**FIGURE 7:**
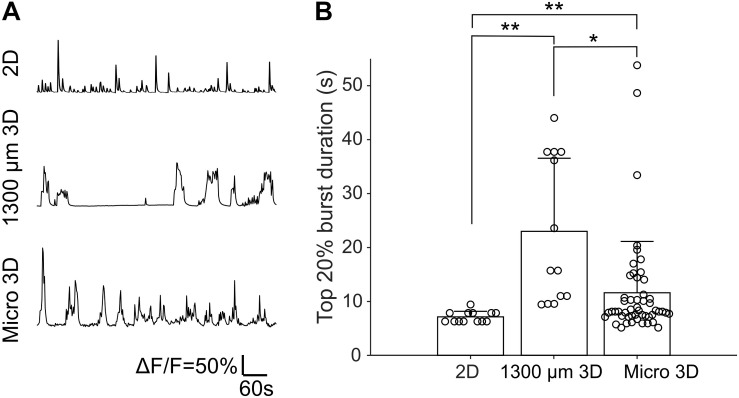
**(A)** Representative Ca^2+^ traces of 2D, 1300 μm 3D and μ3D cultures on DIV 14. **(B)** Top 20% burst durations of 2D, 1300 μm 3D and μ3D cultures on DIV 14. Data for 1300 μm 3D culture has been previously published (Hasan et al., 2019) and is reproduced here for comparison. Data for 2D and μ3D cultures are from experiments described in section “Materials and Methods.” Bars represent the average and error bars are standard deviations. Each circle represents one detected burst. Student’s *t*-tests were used to assess statistical significance. *p < 0.05 and **p < 0.01. Bursts were measured from recordings of 4 cultures for each culture type.

Fluorescence changes due to activity of neurons were observed starting from about DIV 8. As cultures matured, synchronized population bursts were recorded. Dynamic fluorescence changes were recorded every 2 days from DIV 8 to DIV 20. Representative optical recordings are showed in Figure 6A. Cultures became less active on DIV 16-20 and sometimes remained silent during 12 min of optical recording (Figures 6B–E). Burst durations were measured using the method described in data analysis section. Sum of the burst durations in each active recording yielded total active time, which was then divided by the whole recording time for normalization (Figure 6D). Total active times of cultures on DIV 16-20 were significantly shorter (Student’s *t*-test, *n* = 27–41 of active cultures on corresponding DIV, Figure 6B) than earlier DIVs (Figure 6E). We then examined durations of the longest 20% of bursts (Figure 6F). Top 20% burst durations were significantly shorter on DIV 8–10 than DIV 12–20 and they were significantly longer on DIV 20 than DIV 8-14 (Figure 6G, Student’s *t*-test, *n* = 27–41 of active cultures on corresponding DIV, Figure 6B). Waveforms of bursts in μ3D cultures were qualitatively similar (long duration, multi-peak) to bursts in large 3D cultures, but not to shorter, single peak bursts in 2D cultures (Figure 7A). Durations of the longest 20% of bursts were significantly longer in μ3D and large 3D cultures compared to 2D cultures (Figure 7B).

**FIGURE 8:**
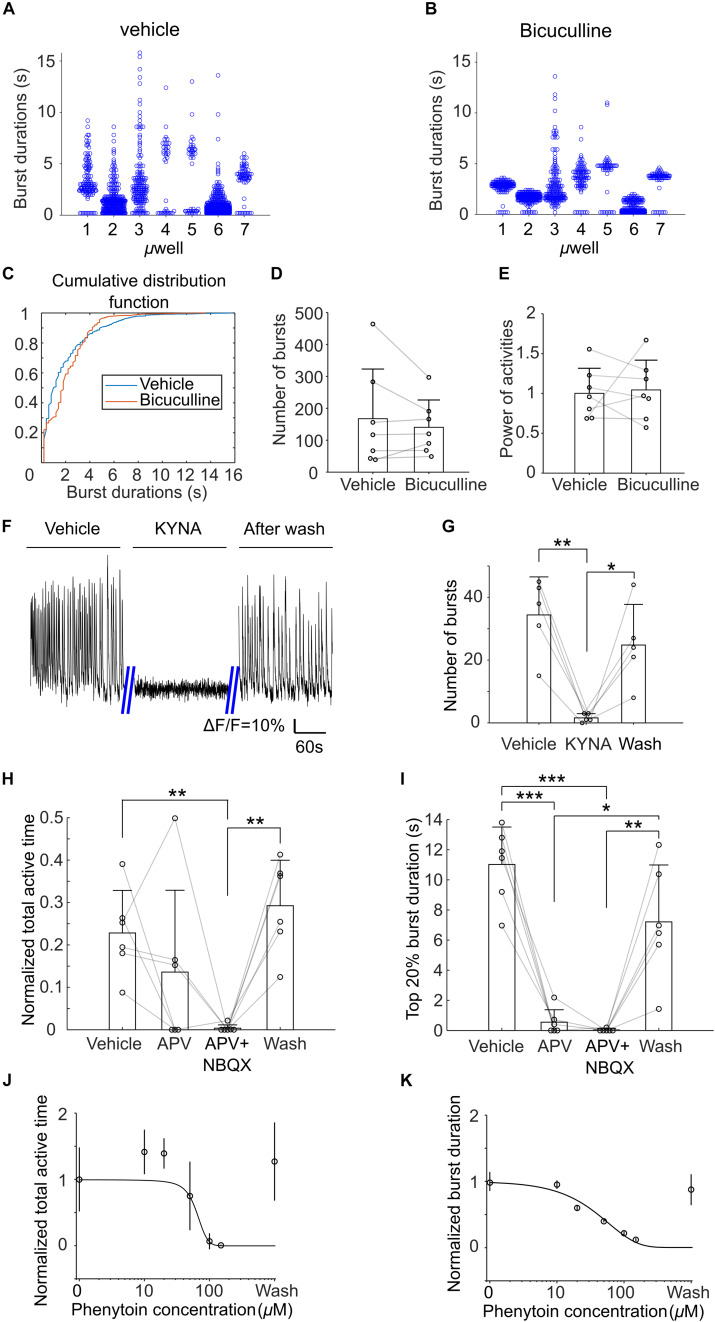
Histograms of burst durations of 7 μ3D cultures in vehicle **(A)** and bicuculline **(B)**. **(C)** Cumulative distribution functions (CDFs) of data in **(A,B)**. Number of total bursts **(D)** and normalized power of activities **(E)** (total areas under bursts, normalized to the average of vehicle) in vehicle and bicuculline. Paired *t*-tests show no significance for either comparison. *n* = 7 cultures, *p* = 0.38 and 0.81 for panel **(D,E)**, respectively. **(F)** Representative recordings of the μ3D culture before, during and after presence of 3 mM KYNA. **(G)** Number of bursts detected before, during and after KYNA application. Paired t tests were used to measure statistical significance. *p < 0.05 and **p < 0.01. *n* = 5 cultures for each condition. Normalized total active time **(H)** and top 20% burst duration **(I)** of μ3D cultures during vehicle, after applying APV, after applying APV + NBQX and after wash. Paired *t*-tests were used to measure statistical significance. *p < 0.05, **p < 0.01, and ***p < 0.001. n = 6 cultures for each condition. Normalized total active time **(J)** and burst duration **(K)** at different concentrations of phenytoin. In panels **(D,E,G–I)**, bars represent the average and error bars are standard deviations. Each circle represents one data point. In panels **(J,K)**, each circle represents the average of 5 cultures and error bars represent 99% confidence interval. Solid lines are curves fitted to sigmoid function y = G/(1 + 10^a1*(con−a2)^) using least absolute residuals (LAR) method, where G = 1 + 10^−a1*a2^, con denotes the concentration of phenytoin. a1 = 3.24 ×10^−2^, a2 = 64.34, *r*^2^ = 0.92, *p* = 2.6×10^−3^ for **(J)** and a1 = 7.40×10^−3^, a2 = −1059, *r*^2^ = 0.96, *p* = 6.08×10^−4^ for **(K).** DIVs of pharmacological experiments: bicuculline on DIV21, kynurenic acid (KYNA) on DIV 19, APV and NBQX on DIV 19 and phenytoin on DIV 18.

Next, we examined effects of bicuculline (on DIV21), kynurenic acid (KYNA, on DIV 19), APV and NBQX (on DIV 19), and phenytoin (on DIV 18) on the activities of μ3D cultures. Application of bicuculline, an antagonist of GABA_A_ receptors (Ueno et al., 1997), decreased the number of both short and long bursts. As shown in Figures 8A,B, after application of bicuculline, durations of bursts in all microwells exhibited more compact clusters centered at middle burst duration (approximately 2–4 s). Cumulative distribution functions (CDFs) of burst duration of vehicle and bicuculline were significantly different (KS test, *p* < 0.001, Figure 8C). The cross of the two CDFs at burst duration of ∼4 s indicates that there were fewer short and long bursts with the presence of bicuculline. On the other hand, number of bursts and normalized power of activity did not change significantly in bicuculline (Figures 8D,E). We found that KYNA, an antagonist of glutamate receptors (Moroni et al., 1988), significantly inhibited activities of the cultures (Figures 8F,G), indicating that spontaneous population bursts in μ3D cultures are dependent on glutamatergic synapses. Presence of APV, an antagonist of NMDA receptors (Morris, 1989), significantly (Figure 8I, paired *t*-test, *p* < 0.001) shortened the average top 20% burst durations without affecting total active time significantly (Figure 8H). Blocking NMDA and AMPA receptors simultaneously with APV and NBQX completely shut down the activities. Curve fittings (Figures 8J,K) show sigmoid relationships of concentration of phenytoin, which is an anti-epileptic drug that acts on voltage-gated sodium channels, to average burst durations and total active time. The activity was completely shut down at 150 μM of phenytoin, and the half effective concentration (EC_50_) of phenytoin is 50 μM.

## Discussion

Spontaneous activity recorded in μ3D cultures consisted of bursts interspersed with quiet periods. Bursts lasted several seconds and contained multiple peaks. Inhibition of bursts by phenytoin indicates that the Ca^2+^ elevations were caused by action potentials, and that Ca^2+^ waveforms can be interpreted as convolution of the population neuronal firing rate with the impulse response of the Ca^2+^ indicator, jRGECO1a (Yaksi and Friedrich, 2006; Hasan et al., 2019). This relationship between dynamic Ca^2+^ in μ3D cultures and population firing rate thus allows us to compare the spontaneous activity in this model to electroencephalogram (EEG) recordings. Qualitatively similar EEG patterns include multi-second bursts of activity separated by quiet inter-burst intervals lasting several seconds, that have been recorded from adult patients (Kroeger and Amzica, 2007; Liley and Walsh, 2013), and from otherwise healthy premature infants (André et al., 2010; Arichi et al., 2017).

In the rat cortex, oscillatory intermittent Ca^2+^ activity, is usually recorded at postnatal days 0–3 (Table 1). These bursts of synchronized activity are termed cortical early network oscillations (cENOs). By postnatal day 8, cENO activity (dependent on glutamatergic synapses) disappears, and cortical giant depolarizing potentials (cGDPs), which are dependent on excitatory GABA synapses, become the dominant synchronized feature (Allene et al., 2008). In our experiments, KYNA (inhibitor of glutamate receptors) reliably blocked spontaneous population activity in μ3D cultures on DIV 19 (Figures 8F,G). Inhibition of both NMDA and AMPA receptors was required to block spontaneous bursts. On the other hand, bicuculline (inhibitor of GABA_A_ receptors) had significant and complex effects on burst waveform, but did not reduce the number of bursts or activity power (Figures 8A–E). This leads us to conclude that spontaneous population bursts in μ3D cultures have the pharmacological profile of cENOs, and not cGDPs, and that developmental stage of neurons in μ3D cultures during period of highest population activity is comparable to rat cortex during the first postnatal week.

Each μ3D culture contains approximately 30 neurons, including around 6 GABAergic neurons on average. Our results show that this number of neurons is sufficient to produce cENO-like intermittent, long burst activity. Interestingly, number of neurons in μ3D cultures falls within size range (5 – 50 neurons) of synchronously active domains in the developing rat cortex (Yuste et al., 1992). Transient, prolonged burst-like elevations in Ca^2+^ were found in small domains of coactive neurons located within ∼100 – 200 μm of each other (Golshani et al., 2009). The size of μ3D cultures is therefore representative of the scale of domains in the developing cortex that generate spontaneous synchronized activity. Unlike *in vivo* cortical domains, networks in μ3D cultures are isolated from the rest of the brain. Thus, each neuron in μ3D culture has only about 30 potential synaptic targets. This likely leads to synaptic scaling: an increase in the strength of synaptic connection between any pair of neurons, which was previously established in 2D *in vitro* cortical networks of varying density (Barral and Reyes, 2016). In μ3D cultures, increased synaptic strength may be the reason that network bursts were triggered by an action potential in a single neuron (Figure 5). This interesting property may make μ3D cultures a useful model for studying mechanisms of burst initiation.

Our results show that 2D cultures have significantly shorter bursts than μ3D cultures. Duration of bursts is directly related to the duration of Ca^2+^ elevations that neurons experience, and may thus significantly impact intracellular signaling where Ca^2+^ plays an important role. Large 3D cultures and μ3D cultures both had significantly longer bursts than 2D cultures, but duration of bursts between two types of 3D cultures was similar (Figure 7). This suggests that it is the spatial arrangement and density of cells, rather than the number of cells in culture (our 2D cultures contained thousands of neurons) are important for generation of prolonged synchronized activity. Dissociated cortex contains astrocytes in addition to neurons; high packing density in 3D cultures may result in closer positioning of astrocytes and neurons compared to 2D. This may influence glutamate cycling and improve neurotransmitter replenishment at the synapses, preventing burst termination due to neurotransmitter depletion (Staley et al., 1998). Other astrocyte-neuron interactions may also be involved. Higher neuronal density may result in high extracellular K^+^ during bursts, which may also play a role in burst prolongation (Chizhov et al., 2018). Neurons and astrocytes may also provide higher trophic support for each other in 3D environment, potentially leading to higher expression of ion channels and pumps, which in turn may also contribute to burst prolongation.

In addition to modeling the size of developing cortical domains and prolonged, intermittent synchronized bursting, μ3D culture method has a practical experimental significance. Pharmacological experiments require continuous, high frame-rate observation of Ca^2+^ for sufficient amount of time to quantify spontaneous bursts before and after drug application. This precludes the use of commercially available high-throughput systems consisting of multiple well plates and a scanning imaging system that observes one culture well at a time. Our approach was to pack many independent cultures into a field of view of an objective with sufficient power to observe fluorescence of the Ca^2+^ indicator. The field of view of a 10x objective is approximately 1 × 1 mm^2^, close to the size of a single well of a commercially available 1536 well plate. To observe multiple cultures in a single field of view, we turned to microfabrication technology to create arrays of μ3D cultures (Figure 2). High density arrays (100 μm culture-to-culture spacing) resulted in axon bridging between individual channels. We thus made lower density arrays with 270 μm spacing to ensure that each μ3D culture is an independent and isolated network. These arrays enabled us to obtain statistically powered results in a single experiment. We determined concentration response of phenytoin using 270 μm arrays as a proof of principle (Figures 8H,I).

## Conclusion

A method of generating μ3D cultures *in vitro* is reported in this work. It was found that μ3D cultures have more complex activity patterns than 2D cultures. μ3D cultures have prolonged, intermittent population bursts with multiple peaks that are similar to human EEG patterns during development or in abnormal brain states. We also demonstrated that spontaneous activities of μ3D cultures were dependent on glutamatergic synapses, similar to cENOs in neonatal rats. The advantages of this method are that: (1) it robustly generates a large number of μ3D cultures in parallel, and (2) microwells can be densely packed so that drug responses of multiple cultures can be monitored simultaneously. This method has the potential to serve as an *in vitro* platform for screening drugs and other compounds against a phenotype of early developmental neuronal activity.

## Data Availability Statement

The raw data supporting the conclusions of this article will be made available by the authors, without undue reservation.

## Ethics Statement

All animal use protocols were approved by the Institution Animal Care and Use Committee (IACUC) at Lehigh University and were conducted in accordance with the United States Public Health Service Policy on Humane Care and Use of Laboratory Animals.

## Author Contributions

YM performed the microfabrication, micro three-dimensional cell cultures, data collection and analysis and co-wrote the manuscript. MH performed the two-dimensional and large three-dimensional cell cultures. ST-L participated in the design of and guided microfabrication, and revised the manuscript. YB guided the experiment design, data collection and analysis, and co-wrote the manuscript. All the authors contributed to the article and approved the submitted version.

## Conflict of Interest

The authors declare that the research was conducted in the absence of any commercial or financial relationships that could be construed as a potential conflict of interest.

## Footnotes

1 http://n2t.net/addgene:100854
